# The lambda sign in gallium‐67 scintigraphy is a useful clue to the early diagnosis of sarcoidosis

**DOI:** 10.1002/ccr3.3295

**Published:** 2020-09-10

**Authors:** Yoshio Hisata, Masaki Tago, Motoshi Fujiwara, Shu‐ichi Yamashita

**Affiliations:** ^1^ Department of General Medicine Saga University Hospital Saga Japan; ^2^ Division of Internal Medicine Nagahama City Kohoku Hospital Shiga Japan

**Keywords:** bilateral hilar lymphadenopathy, diagnosis of sarcoidosis, gallium scintigraphy, lambda sign

## Abstract

A 40‐year‐old man with bilateral hilar lymphadenopathy in chest X‐ray and the λ‐sign in gallium‐67 scintigraphy was subsequently diagnosed with systemic sarcoidosis according to the findings of bronchoalveolar lavage and histopathological examinations of transbronchial lung biopsies. Identifying the λ‐sign could be a valuable clue to the early diagnosis of sarcoidosis.

## Graphical abstract

The λ‐sign strongly suggests the diagnosis of sarcoidosis because all reported patients with the λ‐sign were subsequently diagnosed as having sarcoidosis. Identifying the λ‐sign with gallium‐67 scintigraphy could be a valuable clue to the early diagnosis of sarcoidosis.

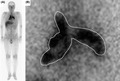

## 1 CASE

**Figure 1:**
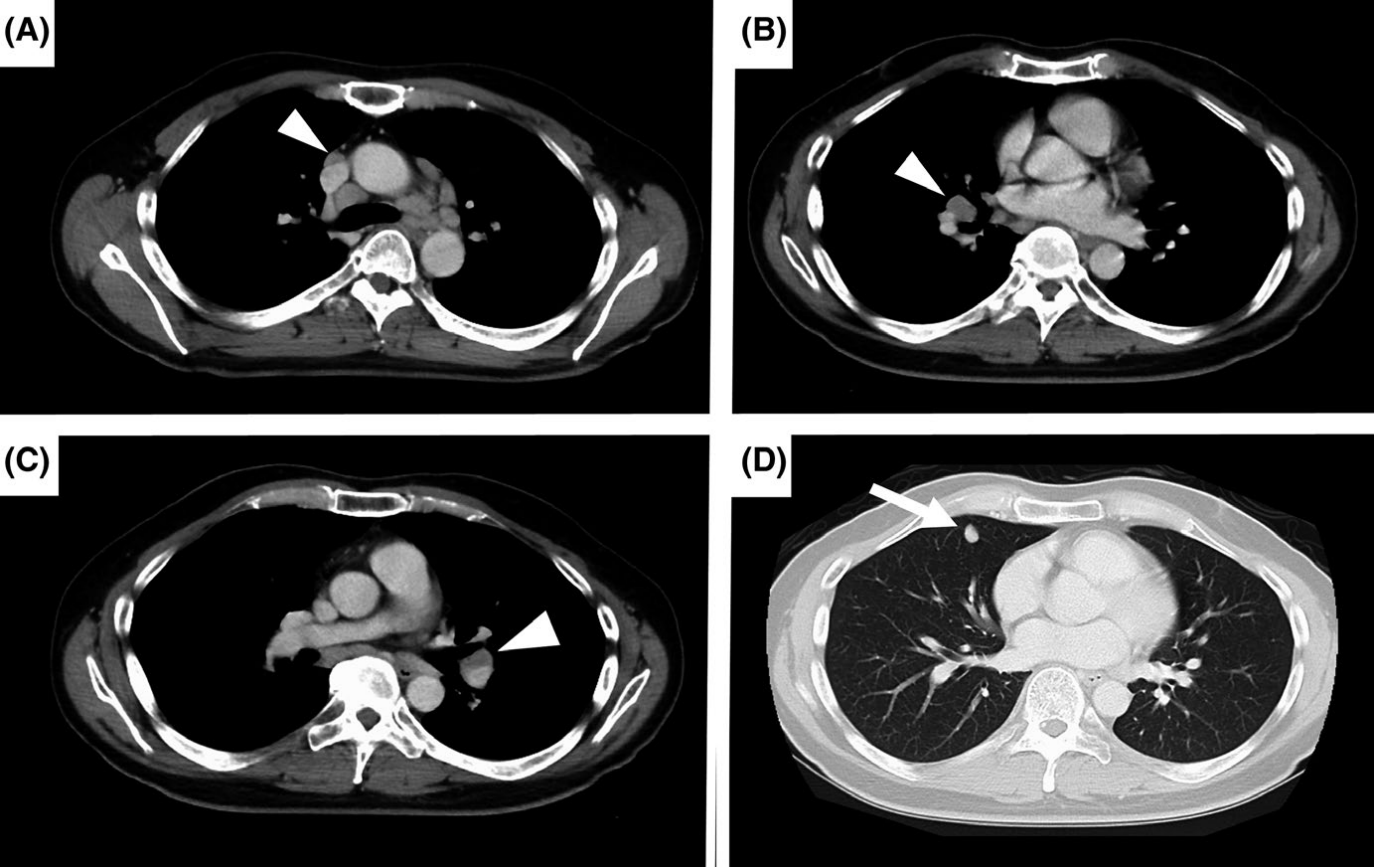
Contrast‐enhanced thoracic computed tomography. (A, B, C) Images of the mediastinal window showing bilateral hilar lymphadenopathy and multiple mediastinal enlarged lymph nodes (arrowheads). (D) Image of the lung‐field window showing a lung nodule in the right S5 region, which was considered to be an intrapulmonary lymph node (arrow)

**Figure 2:**
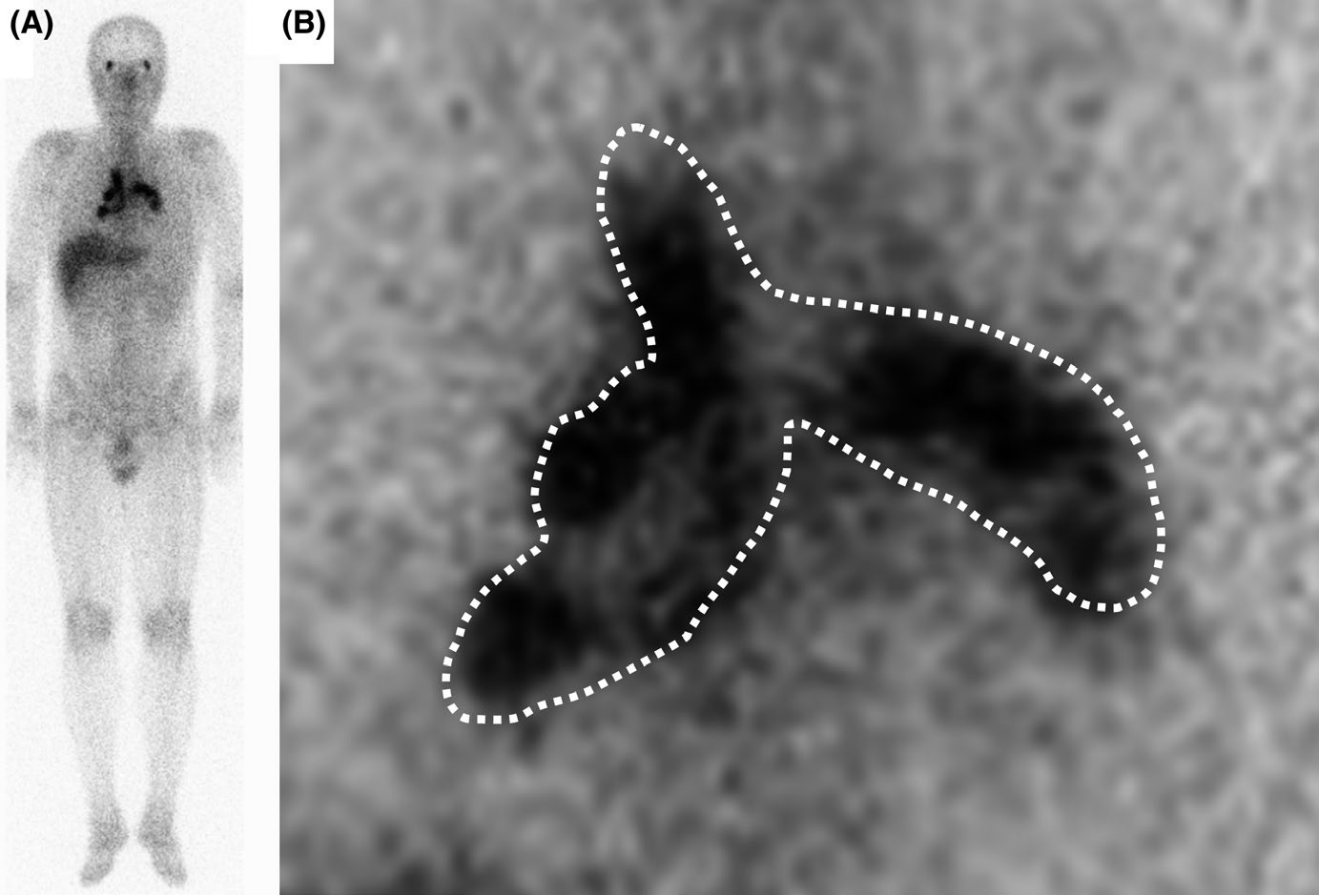
Gallium‐67 scintigraphy. (A) Gallium‐67 scintigraphy shows abnormally high uptake of gallium‐67 in lymph nodes at bilateral hila and mediastinum, and along bronchovascular bundles, which is typical lambda sign, in addition to abnormal uptake in both lachrymal glands. (B)Abnormally high uptake looking like Greek alphabet “λ,” is indicated with dotted line

A 40‐year‐old man visited our hospital with fever, perspiration, fatigue, and joint pain lasting 1 month, with a 5‐kg weight loss. He had neither respiratory symptoms nor visual disturbances. His physical examination findings revealed no abnormality other than tenderness in both ankle joints and nodules in both iridocorneal angles. Blood examinations revealed normal serum calcium and angiotensin‐1‐converting enzyme levels, with no autoantibodies indicating autoimmune diseases. While chest X‐rays showed bilateral hilar lymphadenopathy, contrast‐enhanced thoracic computed tomography revealed hilar lymphadenopathy and enlarged lymph nodes in the mediastinum and at S5 in the right lung field (Figure 1). Gallium‐67 scintigraphy showed abnormally high uptake in lymph nodes at bilateral hila and mediastinum, and along bronchovascular bundles, the typical lambda sign (λ‐sign) (Figure 2). The patient was subsequently diagnosed with systemic sarcoidosis according to the findings of bronchoalveolar lavage and histopathological examinations of transbronchial lung biopsies.

The λ‐sign strongly suggests the diagnosis of sarcoidosis because all reported patients with the λ‐sign were subsequently diagnosed as having sarcoidosis.^1^ Importantly, 33% of patients with no abnormalities on chest X‐ray were reported to have the λ‐sign.^2^ Identifying the λ‐sign with gallium‐67 scintigraphy could be a valuable clue to the early diagnosis of sarcoidosis.

## CONFLICT OF INTEREST

None declared.

## AUTHOR CONTRIBUTIONS

YH: involved in the conception of the study, in the literature search and drafting the study, and clinical care of the patient. MT: involved in the conception of the study, and in the literature search and drafting the manuscript. MF: involved in the literature search and drafting the study. SY: involved in the conception of the study and revising the manuscript.

## ETHICAL APPROVAL

The patient gave permission for the publication of this case report. This manuscript conforms to the provisions of the Declaration of Helsinki in 1995 (as revised in Brazil 2013).
